# Increased Hepatitis E Virus Seroprevalence Correlates with Lower CD4+ Cell Counts in HIV-Infected Persons in Argentina

**DOI:** 10.1371/journal.pone.0160082

**Published:** 2016-07-28

**Authors:** José D. Debes, Maribel Martínez Wassaf, María Belén Pisano, María Beatriz Isa, Martin Lotto, Leonardo G. Marianelli, Natalia Frassone, Estefania Ballari, Paul R. Bohjanen, Bettina E. Hansen, Viviana Ré

**Affiliations:** 1 Department of Medicine, University of Minnesota, Minneapolis, MN, United States of America; 2 Departamento Virología, LACE, Córdoba, Argentina; 3 Instituto de Virología “Dr. J. M. Vanella” Facultad de Ciencias Médicas, Universidad Nacional de Córdoba, Cordoba, Argentina; 4 Hospital Rawson, Cordoba, Argentina; 5 Department of Gastroenterology and Hepatology, Erasmus MC, Rotterdam, the Netherlands; University of Montreal Hospital Research Center (CRCHUM), CANADA

## Abstract

Hepatitis E virus (HEV) is a single-stranded RNA virus that can cause hepatitis in an epidemic fashion. HEV usually causes asymptomatic or limited acute infections in immunocompetent individuals, whereas in immunosuppressed individuals such as transplant recipients, HEV can cause chronic infections. The risks and outcomes of HEV co-infection in patients infected with human immunodeficiency virus (HIV) are poorly characterized. We used a third generation immunoassay to measure serum IgG antibodies specific for HEV in 204 HIV-infected individuals from Argentina and a control group of 433 HIV-negative individuals. We found 15 of 204 (7.3%, 95%CI 3.74–10.96%) individuals in the HIV-positive group to have positive HEV IgG levels suggestive of previous infection, compared to 19 of 433 (4.4%, 95% CI 2.5–6.3%) individuals in the HIV-negative control group (p = 0.12). Among HIV-positive individuals, those with HEV seropositivity had lower CD4 counts compared to those that were HEV seronegative (average CD4 count of 234 vs 422 mm^3^, p = 0.01), indicating that patients with lower CD4 counts were more likely to be HEV IgG positive. Moreover, HEV seropositivity in patients with CD4 counts <200 mm^3^ was 16%, compared to 4.5% in those with CD4 counts >200 mm^3^ (p = 0.012). We found a positive PCR result for HEV in one individual. Our study found that increased seroprevalence of HEV IgG correlated with lower CD4 counts in HIV-infected patients in Argentina.

## Introduction

Hepatitis E virus (HEV) is a single-stranded, non-enveloped RNA virus that can cause hepatitis and is transmitted through an enteric route [1]. In the majority of cases, infection with HEV leads to silent seroconversion or acute self-limited disease [1]. In areas of high endemicity, HEV genoptypes 1 and 2 are common and can present in outbreaks. Particularly severe disease can occur in pregnant women during these outbreaks [1]. Clinically evident infections by HEV genotypes 3 and 4 occur in developed countries as sporadic cases, not associated with outbreaks [2]. When cases occur in immunocompetent individuals, infection with HEV usually leads to silent seroconversion and a chronic course is unlikely [2]. In recent years, however, individuals who are immunosupressed, particularly solid organ transplant recipients, have been shown to develop acute infection, chronic infection or viral reactivation [3, 4].

Although HEV infection is well characterized in immunosupressed solid organ transplant recipients, HEV infection in those with immunosuppression due to other reasons such as human immunodeficiency virus (HIV) infection is less studied. A study from Switzerland showed a 2% seroprevalence of HEV in HIV-infected subjects, but no comparison was made with the immunocompetent population [5]. A recent study in the United States showed 20% seroprevalence of HEV in HIV-infected subjects awaiting liver or renal transplantation [6]. In the following study, we evaluated for the first time the seroprevalence of HEV in HIV-infected persons in central Argentina.

## Methods

### Study population

We analyzed samples from 204 HIV+ persons recruited in Cordoba, Argentina (2010). Of these, 68 were receiving antiretroviral therapy (ART) in a private clinic and were described previously [7], 96 were seen at a different private clinic (58 of them on ART), and 40 were seen at a public hospital for the first time in response to a positive HIV test but were not on ART. Clinical, laboratory and ultrasound information was collected from these subjects at the time of sample collection. A group of 433 HIV-negative subjects described elsewhere [8] was used as a control group. All samples were stored at -80°C before analysis.

### Ethics statements

IRB approval for this study was obtained from the University of Minnesota and Hospital Rawson in Cordoba, Argentina. Written informed consent was obtained from all subjects.

### Enzyme Linked Immunosorbent Assay (ELISA)

IgG and IgM antibodies against HEV were purchased commercially (Diapro, Milan, Italy) and ELISAs were performed strictly following the manufacturer’s instructions. ELISA microplates were coated with HEV-specific synthetic conserved and immunodominant antigens derived from ORF2 and ORF3 of all genotypes. Test results were interpreted as ratio of the sample (S) and the cut-off (CO) (S/CO). Samples with ratio bellow 0.9 were considered negative, between 0.9–1.1were equivocal, and above 1.1 were considered positive results.

### Polymerase Chain Reaction (PCR)

Nested-PCR was performed targeting ORF 2, following protocols previously described [8, 9]. PCR products (10 μL) were analyzed by electrophoresis using TBE buffer and a 2% agarose gel containing 0.5 g/mL of ethidium bromide, and visualized under UV light. The lower limit of detection of the nested PCR was 31.6 PID (pig infectious dose) [9].

### Statistical analysis

When comparing variation of prevalence, students t-tests and Fisher exact test were used. P values <0.05 were considered significant. Logistic regression was used to compute the odds ratio for CD4 adjusted for age. The chi-square test was used when analyzing CD4 cut offs.

## Results

**Fig 1:**
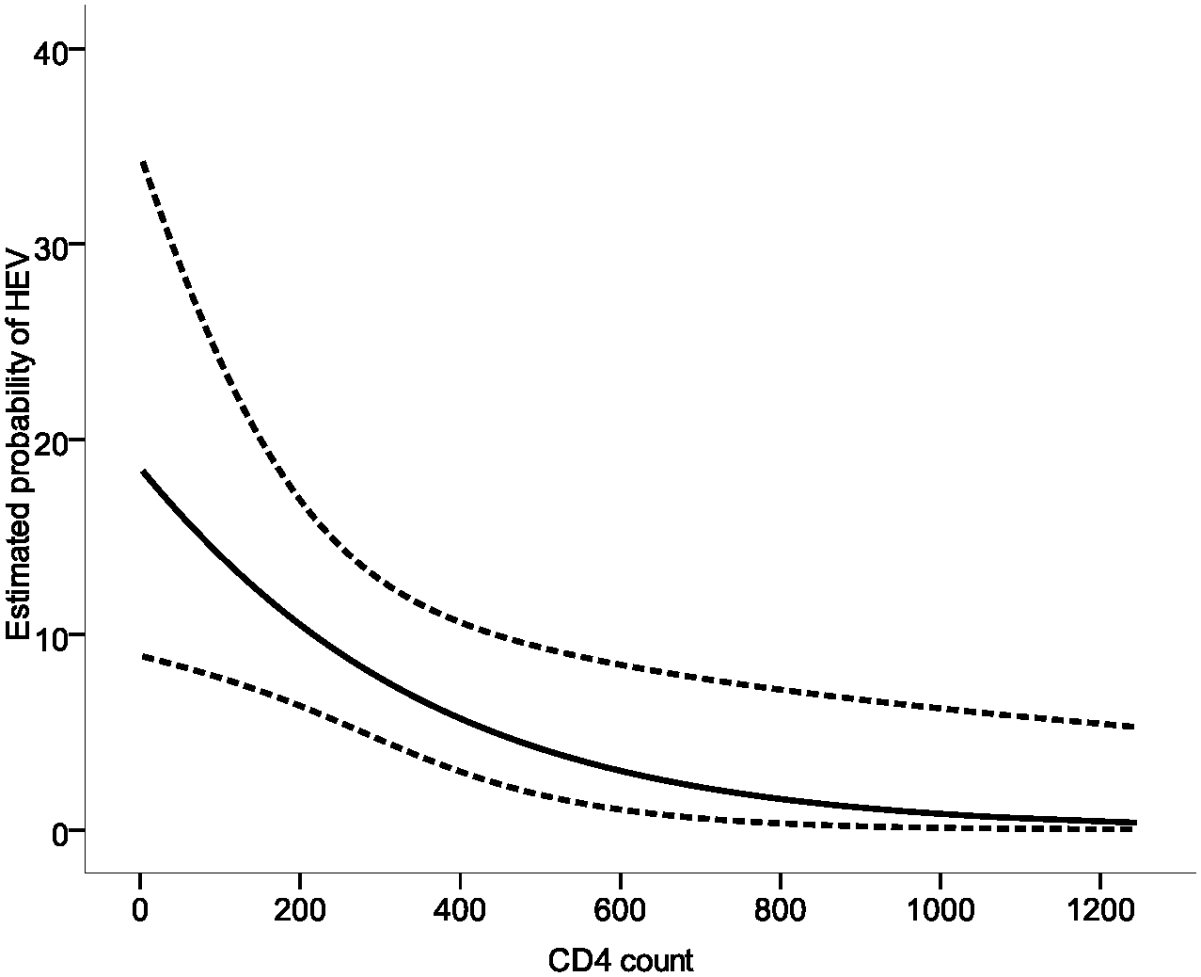
Estimated probability of HEV by CD4 count (full line) with 95% confidence interval (dotted lines).

In our cohort of 204 HIV-positive subjects, 15 (7.35%, 95% CI 3.74–10.96%) were positive for HEV IgG in serum. In our previous study in 433 HIV-negative subjects at the same research site, the prevalence of HEV was 4.4% (95% CI 2.5–6.3%,19/433, p = 0.12) [8]. The mean age of HEV-positive patients was 44.9 years versus 39.7 years in those HEV-negative (p = 0.11, not significant). We compared CD4 counts for the HIV-positive subjects who were HEV IgG positive versus negative. The group of HIV-infected subjects that were seropositive for HEV had lower CD4 counts when compared to those that were seronegative for HEV (average count of CD4/mm^3^ 234 vs 422, p = 0.01). Based on logistic regression analysis the coefficient for CD4 count was significant, Z = -2.44, p = 0.01, with an odds ratio lower than 1: 0.967 (95% CI: 0.942–0.994) for a CD4 increase of 10 counts, indicating that individuals with low CD4 counts were more likely to be HEV seropositive (Fig 1). The higher risk for HEV seropositivity with decreasing CD4 count remained significant after adjusting for age (Z = -2.69, p = 0.006, OR = 0.969, 95% CI: 0.943–0.995).

When we divided patients among those with severe immunosuppression, defined as CD4 counts <200 mm^3^ versus those with CD4 counts >200 mm^3^, we found that seropositivity for HEV IgG was 16% (95% CI 5.48–26.5%) versus 4.5% (95% CI 1.22–7.87%) respectively (p = 0.012). The median age for both groups (seropositive and seronegative) was similar: 39 years for those HEV seropositive (IQR 35–41) and 38 years (IQR 31–46) for those HEV seronegative (Table 1). We found no difference in HEV seroprevalence among those on ART or not on ART (8.4% versus 6.4%, p = 0.59).

**Table 1:** HEV seroprevalence according to CD4 count cut off.

		**HEV IgG**		**Odds Ratio of CD4**	
**Cohort**	**Cohort Size**	**Seropositive**		*by increase 10 counts*,	**p-value**
	**N**	**n**	**prevalence, 95% CI (%)**	**95% CI**	*log-Likelihood*
**Total**	204	15	7.35	0.967	0.006
			(3.74–11.96)	(0.942–0.994)	
				**Odds Ratio of CD4** *<200 versus ≥200 mm/*^*3*^,	**p-value**
	**N**	**n**	**prevalence, 95% CI (%)**	**95% CI**	*log-Likelihood*
**Total**					
CD4<200 mm/^3^	50	8	16.00 (5.48–26.52)	4.00 (1.37–11.67)	0.012
CD4≥200 mm/^3^	147	7	4.55 (1.22–7.87)	1 (reference)	

OR: Odds Ratio; CI: Confidence Interval

Five subjects that were positive for HEV IgG were also positive for HEV IgM, suggesting acute or recent infection. We had access to clinical information for only one of these five individuals; this person had normal liver enzymes and no gastrointestinal symptoms at the time of sample collection. HEV RNA detection through PCR was positive in only one of the 204 samples from HIV+ subjects. This sample belonged to a subject with a CD4 count of 29/mm^3^ who was not on ART. Interestingly, this subject had symptoms of nausea and vomiting but had negative HEV IgG and IgM ELISA tests, suggesting that this could have represented a case of acute HEV infection where seroconversion had not yet occurred.

## Discussion

Our study shows that HIV-positive subjects in central Argentina have a higher but not statistically significant seroprevalence of HEV when compared to HIV-negative controls. In HIV-infected subjects with low CD4 counts, the seroprevalence was significantly higher than in HIV-infected subjects with high CD4 counts or HIV-negative subjects.

Our group recently performed a study on HEV infection in immunocompetent persons in central Argentina and found a seroprevalence of 4.4% for HEV IgG [8]. In other studies, the seroprevalence of HEV antibodies varies among immunocompetent and immunosuppressed subjects [2]. The increased seroprevalence of HEV in immunosupressed populations is of importance since immunosuppressed individuals, particularly those that have undergone solid-organ transplantation, can develop chronic infection with HEV [4]. Hering et al. found 15% HEV seroprevalence among renal transplant recipients, and 10% of these subjects had positive HEV RNA, suggesting they had chronic infection with persistent viral replication [10]. Very few samples in that study had both positive HEV IgG and HEV RNA, and therefore acute infection could not be excluded. The prevalence of HEV in transplant recipients varies depending on geographical location. In France 13% to 39% of renal and liver transplant recipients were positive for HEV IgG, while this number was 2% and 2.9% in liver and transplant recipients in the Netherlands and Germany, respectively [4, 11, 12].

Although most studies suggest an increased prevalence of HEV in immunosuppressed subjects, as well as a higher risk of chronic disease, little is known about the effect of HIV on HEV infection and results are mixed across different studies. Kenfak-Foguena et al. reported a seroprevalence of HEV of 2.6% in HIV-infected subjects in Switzerland. Interestingly, this study was performed on subjects with unexplained elevated liver enzymes, in which a higher seroprevalence of HEV might have been expected [5]. Sherman et al. found an HEV seroprevalence of 20% in HIV-infected subjects awaiting solid-organ transplant. The study found no active infection, based on RNA levels of HEV, in any of the subjects. In that study, patients had well-controlled HIV viremia, as expected in a cohort waiting for solid organ transplantation [6]. Pineda et al. reported a seroprevalence of HEV IgG of 26% in an HIV-infected population in Spain [13].

Some of the differences in HEV IgG prevalence across studies can be attributed to differences in the commercially available assays. While initial assays showed a very high number of false positives, newer tests, including the one used in our study, have shown to have good sensitivity and specificity for detecting HEV IgG antibodies [14]. Caution is necessary when interpreting results from immunoglobulin assays in the setting of humoral or cellular immunosuppression, since the assay performance might not be optimal.

Overall, studies addressing HEV prevalence in HIV infected subjects have been cross-sectional and performed on individuals with controlled HIV disease. In our study, although there was a difference of HEV IgG seroprevalence in HIV-positive persons compared to HIV-negative controls, this difference was significant only in the subgroup of subjects with low CD4 counts. This difference was seen regardless of whether or not subjects were receiving ART. Our results are consistent with Pineda et al., who found increased seroprevalence of HEV in HIV-infected subjects in Spain with CD4 counts below 500/mm^3^ when compared to those CD4 counts above 500/mm^3^ [13]. It might be expected that detection of IgG would have lower sensitivity in HIV-infected subjects with lower CD4 counts, and therefore, more profound immunosuppression, but we found higher seroprevalence in the subgroup with the lowest CD4 counts. It appears, however, that marked immunosuppression, indicated by lower CD4 counts, may predispose subjects to infection with HEV.

There might be differences in the socio-economic status of our HIV-infected subjects from different clinics. Although we did not use a specific questionnaire to address these differences, some subjects were seen in a private hospital (likely of higher income), while other subjects were seen in a public hospital (likely of lower income). In our previous study in immunocompetent subjects, we found a difference in HEV seroprevalence based on socioeconomic status [8]. Since HEV is transmitted in a fecal-oral route, it might be expected to be more prevalent in populations where hygiene standards are lower.

In those subjects positive for HEV IgG in which we had clinical information available (n = 4), liver enzymes were normal and none showed clinical evidence of chronic hepatitis, but all had some abnormality on liver US, such as increased size (n = 3) or increased echogenicity (n = 3). RNA levels were positive in only one sample from the entire cohort of samples. This subject had symptoms of nausea, vomiting and diarrhea, suggesting an early acute infection with HEV.

The clinical significance of positive HEV IgG in our population is unclear. With reports indicating higher likelihood of chronic infection in subjects with solid organ transplant, we believe studies are necessary to perform longer follow-up of subjects with HIV and HEV to better characterize the chronicity of HEV infection in HIV-infected patients, particularly since HEV can now be treated with ribavirin [15].

In summary, this study shows a higher prevalence of HEV IgG in HIV-positive subjects with low CD4 counts. Studies evaluating a larger number of HIV-infected subjects, including those receiving and not receiving ART, are necessary to further understand this finding and its clinical significance.

## Supporting Information

S1 File Minimal data set Debes et al. (XLSX)
